# Effect of acute TLR4 inhibition on insulin resistance in humans

**DOI:** 10.1172/JCI162291

**Published:** 2022-11-01

**Authors:** Hanyu Liang, Nattapol Sathavarodom, Claudia Colmenares, Jonathan Gelfond, Sara E. Espinoza, Vinutha Ganapathy, Nicolas Musi

**Affiliations:** 1 Barshop Institute for Longevity and Aging Studies and; 2 Diabetes Division, Department of Medicine, UT Health San Antonio, San Antonio, Texas, USA.; 3 San Antonio Geriatric Research, Education and Clinical Center, Audie L. Murphy VA Medical Center, San Antonio, Texas, USA.; 4 Department of Population Health Science, UT Health San Antonio, San Antonio, Texas, USA.

**Keywords:** Endocrinology, Metabolism, Diabetes, Obesity

## Abstract

**Background:**

Studies in cell cultures and rodents suggest that TLR4 is involved in the pathogenesis of insulin resistance, but direct data in humans are limited. We tested the hypothesis that pharmacologic blockade of TLR4 with the competitive inhibitor eritoran would improve insulin resistance in humans.

**Methods:**

In protocol I, 10 lean, healthy individuals received the following 72-hour i.v. infusions in a randomized crossover design: saline (30 mL/h) plus vehicle; Intralipid (30 mL/h) plus vehicle; or Intralipid (30 mL/h) plus eritoran (12 mg i.v. every 12 hours). In protocol II, also a randomized crossover design, 9 nondiabetic individuals with obesity received eritoran or vehicle for 72 hours. The effect of eritoran was assessed with euglycemic hyperinsulinemic clamps.

**Results:**

In protocol I, lipid infusion significantly decreased peripheral insulin sensitivity (M value) by 14% and increased fasting plasma glucose (FPG) concentrations, fasting plasma insulin (FPI) concentrations, and the homeostatic model assessment of insulin resistance (HOMA-IR) index by 7%, 22%, and 26%, respectively. Eritoran did not prevent lipid-induced alterations of these metabolic parameters. Eritoran also failed to improve any baseline metabolic parameters (M, FPG, FPI, HOMA-IR) in individuals with obesity and insulin resistance (protocol II).

**Conclusions:**

Acute TLR4 inhibition with eritoran did not protect against lipid-induced insulin resistance. Short-term eritoran administration also failed to improve obesity-associated insulin resistance. These data do not support a role for TLR4 in insulin resistance. Future studies with a different class of TLR4 inhibitors, longer drug exposure, and/or lipid-enhancing interventions richer in saturated fats may be needed to further clarify the role of TLR4 in metabolic dysfunction in humans.

**Trial registration:**

ClinicalTrials.gov NCT02321111 and NCT02267317.

**Funding:**

NIH grants R01DK080157, P30AG044271, P30AG013319, and UL1TR002645.

## Introduction

Insulin resistance is one of the earliest abnormalities in the pathogenesis of type 2 diabetes mellitus (T2DM). Therefore, interventions designed to improve insulin resistance are likely to be effective in preventing and treating T2DM. However, the pathophysiology of insulin resistance is not fully understood. A large body of evidence suggests that activation of TLR4 may be a key mechanism underlying the inflammatory state and insulin resistance of obesity and T2DM (1–3). TLRs are cell-surface receptors that generate immune responses to pathogens by activating a cascade of inflammatory events. TLR4, one of the best-characterized TLRs, is highly expressed in immune cells and insulin target cells, including myocytes, adipocytes, and hepatocytes. Lipopolysaccharide (LPS or endotoxin), an outer membrane component of Gram-negative bacteria, is the main natural ligand of TLR4. Saturated fatty acids (FAs) contained in the lipid A moiety of LPS are essential for its biologic activity (4). In addition, saturated free fatty acids (FFAs) from a nutritional or metabolic source can function as TLR4 agonists (4).

Upon stimulation of TLR4 and its coreceptors CD14 and MD-2, the adaptor proteins TIRAP and MyD88 are recruited to the Toll/IL-1 receptor (TIR) domain of TLR4 (5). This association leads to the activation of proinflammatory kinases, including the MAPKs; JNK, p38, and ERK; and the IκB kinase (IKK) complex, which results in the activation of transcription factors such as NF-κB (5). Accordingly, inhibition of the JNK or IKKβ/NF-κB pathways can improve insulin sensitivity (6, 7).

Disrupted TLR4 function protects against acute and chronic fat-induced impairments in insulin action in mice (1, 2, 8). In general, studies have shown a protective effect of TLR4 inhibition against lipid-induced insulin resistance in skeletal muscle (1). Some (1), albeit not all (9) mouse studies that investigated the effect of TLR4 disruption also have revealed protection at the level of the liver. We and others have shown that insulin-resistant individuals have increased TLR4 expression and signaling in skeletal muscle (10) and peripheral mononuclear cells (11, 12). Moreover, plasma concentrations of the TLR4 ligands FFA (13) and LPS (14–16) are often elevated in those with insulin resistance. Systemic LPS elevation associated with metabolic dysfunction (i.e., metabolic endotoxemia) is thought to result from intestinal barrier dysfunction (17). Overall, these findings suggest that activation of TLR4 may be a key mechanism underlying the inflammatory state and insulin resistance in obesity and T2DM. Yet, no direct data exist to indicate a role for TLR4 on metabolic dysfunction in humans. In this study, we examined the effects of a selective TLR4 antagonist to further evaluate the role of TLR4 in the etiology of insulin resistance in humans. To our knowledge, this is the first human study to examine how TLR4 inhibition affects whole body glucose metabolism.

To inhibit TLR4, we used eritoran (E5564, Eisai Pharmaceuticals), a structural analog of lipid A (18). Eritoran was developed as an i.v. drug to treat sepsis. It competitively and selectively binds to TLR4-MD2 and prevents LPS from initiating an inflammatory response (18–21). The pharmacokinetic and pharmacodynamic properties and safety profile of eritoran have been studied extensively during phase 1, 2, and 3 experimental studies of endotoxemia in healthy volunteers and patients with severe sepsis (21–24). Eritoran, which has a half-life of 25–30 hours, is well-tolerated and has no significant toxicity (21–24). We previously showed that eritoran mitigates lipid-induced insulin resistance in rats (3).

## Results

### Effect of eritoran on lipid-induced insulin resistance (protocol I).

**Figure 1:**
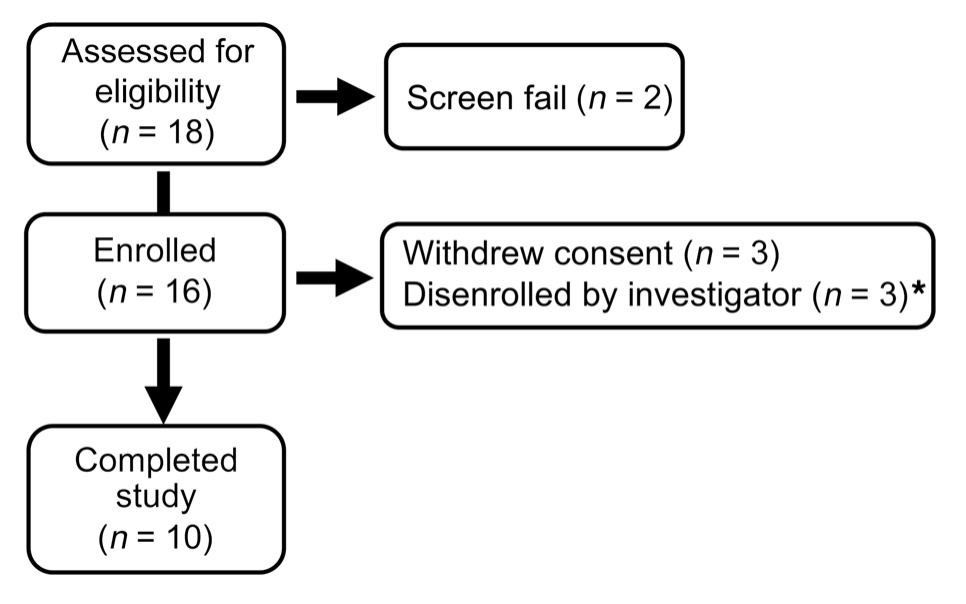
Protocol I flowchart. *One individual had hypertriglyceridemia during lipid infusion; 1 individual had anemia attributed to blood draws; and 1 individual had excessive weight loss during the study period.

The study flowchart and participants’ characteristics are shown in Figure 1 and Table 1, respectively. Study participants were lean and had normal glucose tolerance. They did not have systemic diseases and were not taking any medications known to affect glucose metabolism. Individuals were admitted to the Audie L. Murphy VA Hospital in San Antonio, Texas, to undergo the following 72 hour long i.v. infusions (randomly), separated by 4–8 weeks: saline (30 mL/h)+vehicle every 12 hours; 20% Intralipid (30 mL/h)+vehicle; or 20% Intralipid (30 mL/h)+eritoran (12 mg every 12 hours). Eritoran or vehicle administration began 2 hours before the saline or lipid infusions.

**Figure 2:**
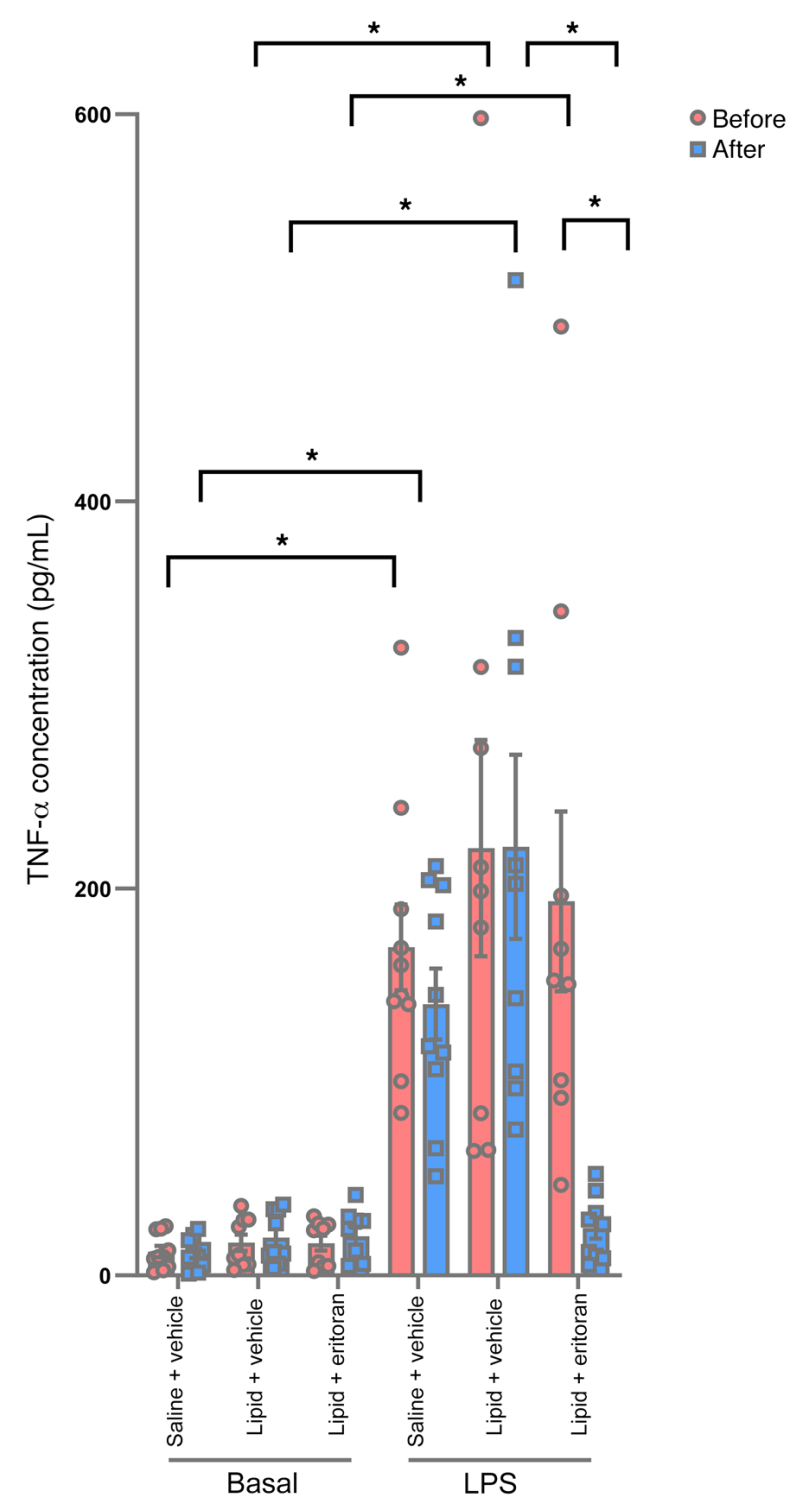
Effect of eritoran on ex vivo LPS–stimulated TNF-α release in peripheral blood isolated from lean individuals before and after saline+vehicle, lipid+vehicle, or lipid+eritoran infusions. Peripheral blood was treated with LPS for 3 hours, and plasma TNF-α concentrations were measured by ELISA. All values indicate the mean ± SEM. *P < 0.05, by 2-way ANOVA followed by Holm-Šídák’s multiple-comparison test.

To determine the pharmacodynamic activity of eritoran, we added LPS to peripheral whole blood obtained from participants before and after infusions. Blood was stimulated with 10 ng/mL LPS ex vivo and assayed for the release of TNF-α into the plasma. As expected, we found that TNF-α concentrations were markedly increased after LPS stimulation in the increased after LPS stimulation in the saline+vehicle and lipid+vehicle groups (Figure 2). (Figure 2). This effect was completely inhibited in the lipid+eritoran group, demonstrating that eritoran is a potent systemic inhibitor of TLR4.

**Figure 3:**
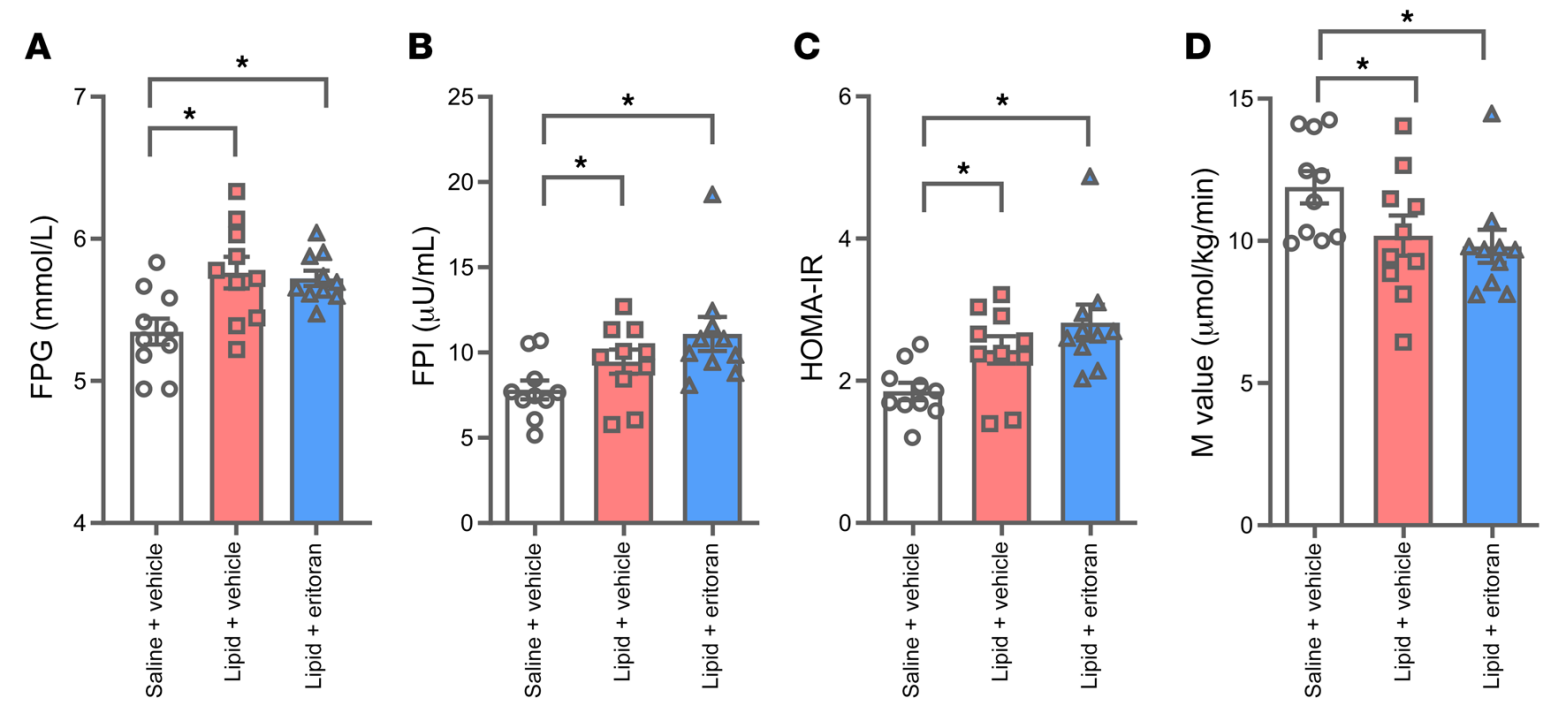
Effect of lipid and eritoran on metabolic parameters in lean individuals. FPG (**A**) and FPI (**B**) levels, the HOMA-IR index (**C**), and M values (**D**) were determined following saline+vehicle, lipid+vehicle, and lipid+eritoran infusions. All values indicate the mean ± SEM of data obtained from 10 individuals. *P < 0.05, by 1-way ANOVA followed by Holm-Šídák’s multiple-comparison test.

At the end of the 72-hour infusions, we measured insulin sensitivity using euglycemic, hyperinsulinemic clamps. Compared with saline+vehicle, the lipid+vehicle infusion significantly impaired several parameters of glucose metabolism, including increasing fasting plasma glucose (FPG; 7%) and fasting plasma insulin (FPI; 22%) concentrations and the homeostatic model assessment of insulin resistance (HOMA-IR) index (26%), and reducing peripheral insulin sensitivity (M value; 14%) (Figure 3). Lipid+eritoran infusion also significantly increased FPG (6%) and FPI (42%) levels and the HOMA-IR index (47%), and lowered M values (18%) compared with saline+vehicle (Figure 3). We observed no statistically significant differences in these parameters between lipid+eritoran and lipid+vehicle treatments. Supplemental Figure 1 shows individual metabolic outcomes and how they trended according to the different interventions.

### Effect of eritoran on obesity-associated insulin resistance (protocol II).

**Figure 4:**
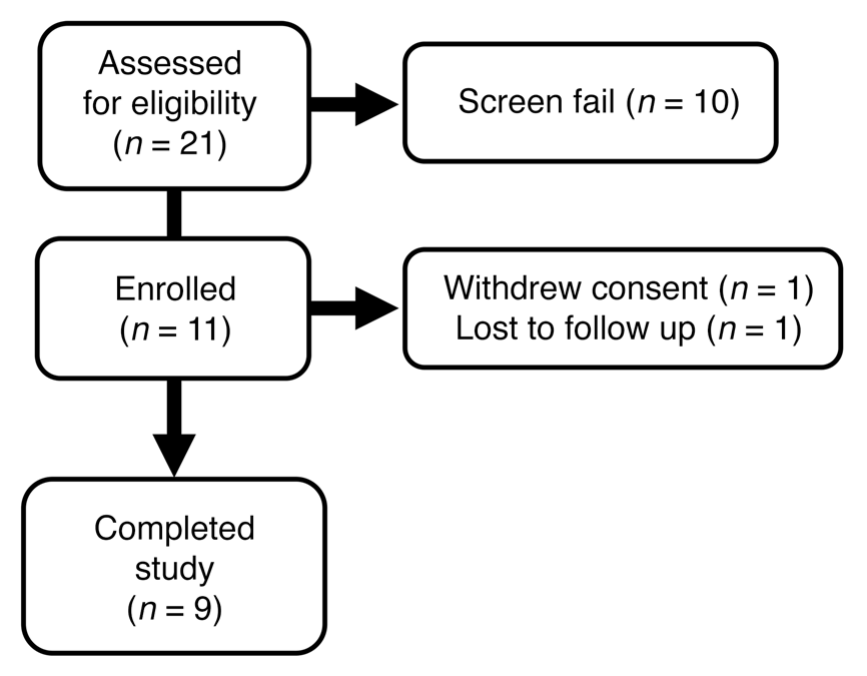
Protocol II flowchart.

**Figure 5:**
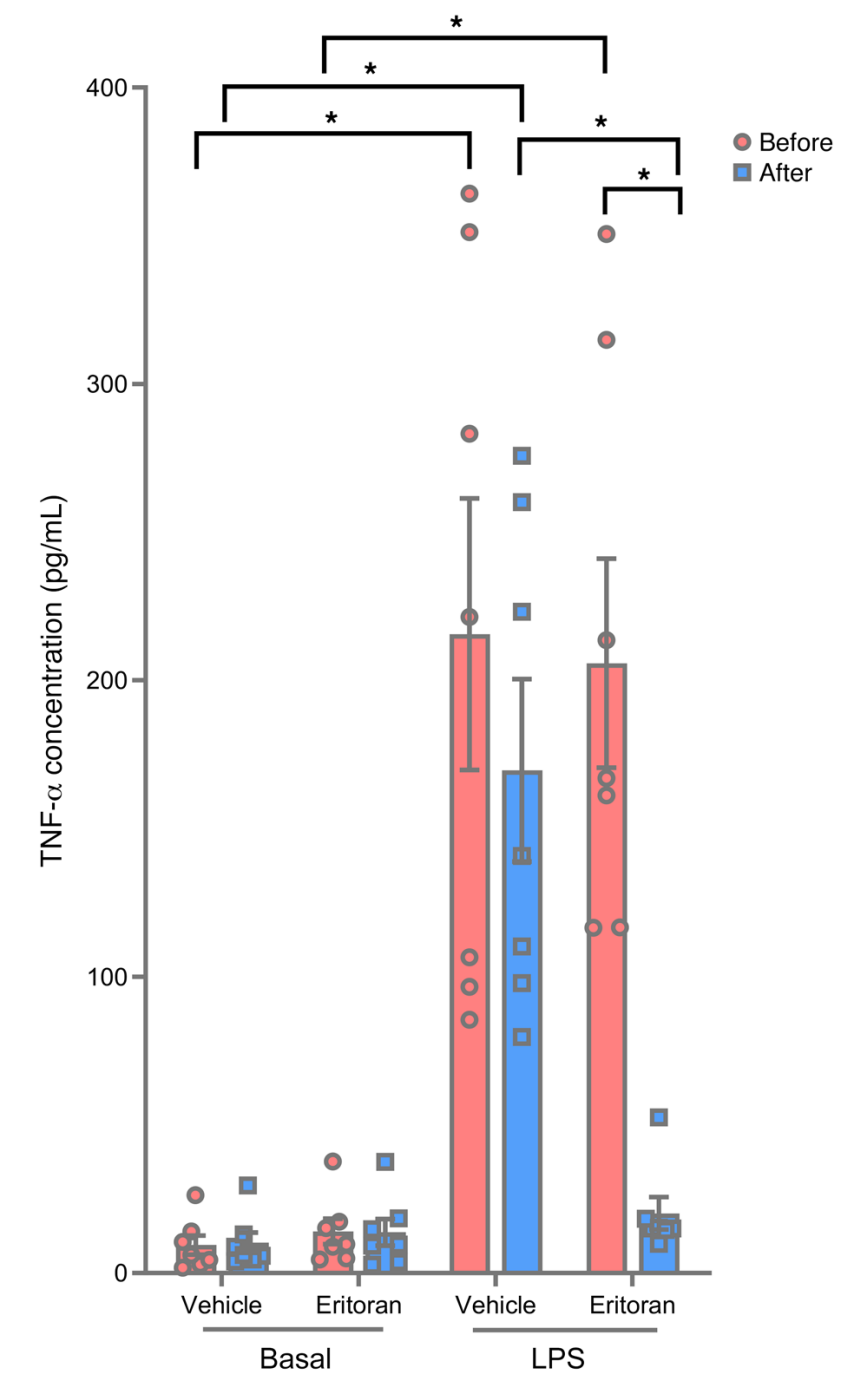
Effect of eritoran on ex vivo LPS–stimulated TNF-α release in peripheral blood isolated from individuals with obesity before and after vehicle or eritoran administration. Peripheral blood was treated with LPS for 3 hours, and plasma TNF-α concentrations were measured by ELISA. All values indicate the mean ± SEM. *P < 0.05, by 2-way ANOVA followed by Holm-Šídák’s multiple-comparison test.

In protocol II, we evaluated whether blocking TLR4 with eritoran reverses baseline measures of insulin resistance in nondiabetic individuals with obesity. The study flowchart is shown in Figure 4, and the characteristics of these study participants are shown in Table 2. Participants had normal glucose tolerance, did not have systemic diseases, and took no medications known to affect glucose metabolism. They were admitted to the hospital on 2 occasions, separated by 4–8 weeks, to receive (randomly) eritoran 12 mg i.v. or vehicle every 12 hours for 72 hours. As in protocol I, ex vivo LPS stimulation testing in whole blood showed that eritoran robustly inhibited TNF-α production in individuals with obesity (Figure 5).

**Figure 6:**
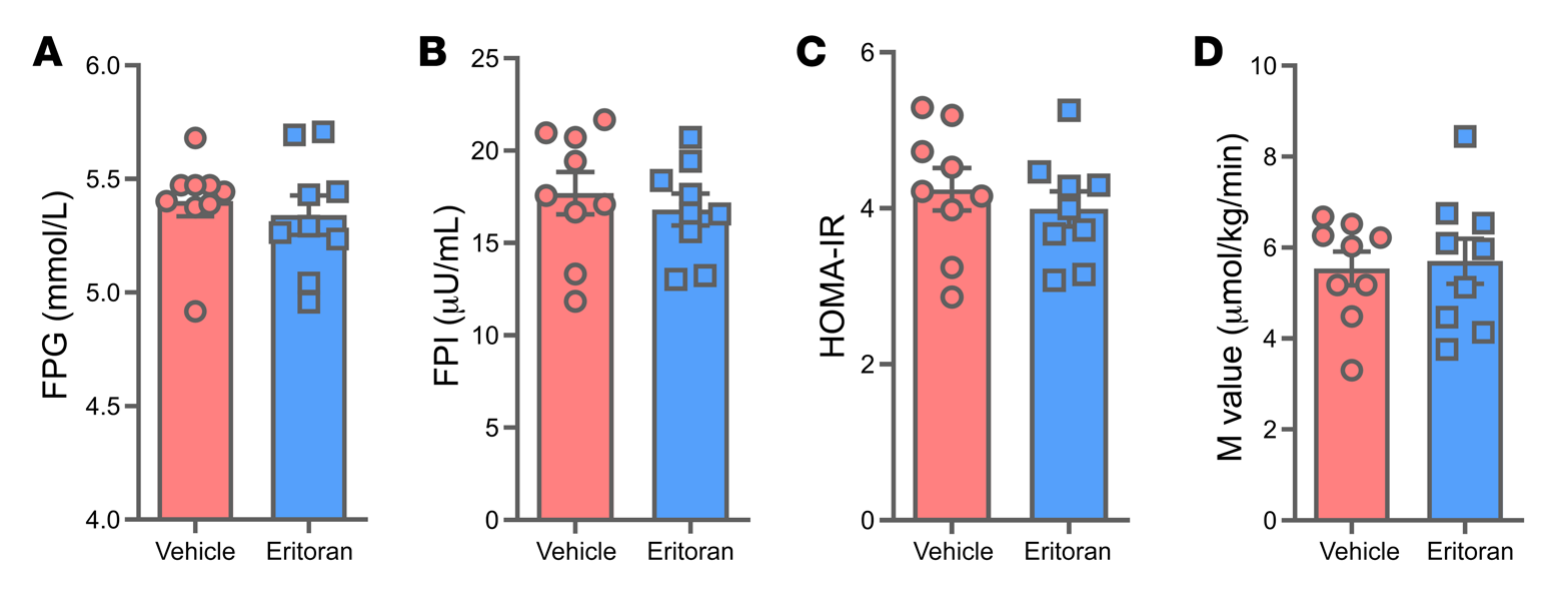
Effect of eritoran on metabolic parameters in individuals with obesity. FPG (**A**) and FPI (**B**) concentrations, the HOMA-IR index (**C**), and M values (**D**) were determined following vehicle or eritoran administration. All values indicate the mean ± SEM of data obtained from 9 participants. Comparisons were made using a paired *t* test or a Wilcoxon signed-ranked test.

Insulin sensitivity was measured with insulin clamps at the end of the eritoran or vehicle treatments. Compared with the lean participants who received saline+vehicle (from protocol I), baseline (after vehicle) FPI and HOMA-IR were elevated, and peripheral insulin sensitivity (M) was reduced in individuals with obesity (Supplemental Figure 3). Baseline LPS and TNF-α concentrations were significantly higher in individuals with obesity, indicative of an inflammatory state (Supplemental Figure 3). Nonetheless, eritoran (versus vehicle) had no effects on metabolic parameters in the group of participants with obesity (Figure 6 and Supplemental Figure 2).

### Tolerability and safety.

Eritoran was well tolerated; it did not cause toxicity or any serious adverse events. Transient, moderate phlebitis at the eritoran infusion site was observed in 4 of 10 individuals from protocol I and in 3 of 9 individuals from protocol II.

## Discussion

Since cardiometabolic diseases are characterized by a low-grade inflammatory state, therapies that target inflammatory pathways could be helpful. However, drugs that target inflammation mediators such as IL-1β (25, 26) and TNF-α (27) have shown minimal improvements in glucose metabolism in clinical studies, highlighting the need to develop and evaluate interventions that target different mechanisms underlying inflammation. Preclinical studies have established TLR4 as an important mechanistic link between chronic low-grade inflammation and insulin resistance (28). Thus, in this study, we used a TLR4 inhibitor to directly examine the role of TLR4 on insulin resistance in humans. We postulated that, if positive, findings from this proof-of-concept study could spur future investigations to develop agents with favorable dosing and routes of administration and to optimize the long-term safety and efficacy of TLR4 inhibitors.

The lipid infusion reduced the M value and increased the HOMA-IR index, indicative of impaired insulin sensitivity in the muscle and liver, respectively. However, contrary to our hypothesis, TLR4 inhibition with eritoran did not prevent lipid-induced insulin resistance. These results are in contrast to studies in rodent models of high-fat diet–induced metabolic dysfunction, in which TLR4 deficiency was beneficial (1). This discrepancy may involve differences in physiology and metabolism between species, as well as differences in study design, duration of lipid exposure, and lipid composition. Most studies of high-fat feeding in rodents have used 60% saturated fat diets (1, 29, 30) or lard oil infusion (2). In contrast, the lipid infusion we used contained only 16% saturated lipid. The relative proportion of saturated lipids could be an important factor because saturated, but not unsaturated, FFAs activate TLR4 signaling (4). Indeed, work from Davies et al. suggests that protection against insulin resistance in TLR4-deficient mice seemed to occur selectively when the mice consumed a diet high in saturated lipids (31). Moreover, other work suggests that TLR4 may not function as a receptor for saturated FAs (32) and that fetuin-A acts as a TLR4 endogenous ligand that mediates lipid-induced insulin resistance (33).

Mechanisms by which lipids induce insulin resistance may be independent of TLR4, such as intracellular accumulation of lipid metabolites (34). Diacylglycerols are thought to be an important mediator of insulin resistance (35–38) via activation of PKCθ and PKCδ (35–38). Ceramides can impair insulin action, presumably through activation of PKCζ (39). In addition, levels of long-chain fatty acyl-CoA and acylcarnitines are also elevated in insulin-resistant individuals and may adversely impact insulin sensitivity and glucose metabolism (34, 40, 41).

Eritoran increases survival in animal models of bacterial sepsis (18), but it did not improve clinical outcomes in a phase III clinical trial in patients with septic shock (42). Here, we used eritoran to investigate the role of TLR4 in insulin resistance and confirmed that it was a potent TLR4 inhibitor in peripheral blood from the research participants who received the drug. Yet its pharmacokinetic properties may be a limitation. Because eritoran was developed for i.v. administration, it cannot be given chronically to humans, and TLR4 inhibitors may require long-term dosing to significantly impact insulin action in humans. Eritoran has a volume of distribution of 40–55 mL/kg (43), and its muscle tissue penetration and target engagement in humans have not been determined. It is also possible that other TLR4 inhibitors with a different mechanism of action may be more effective against insulin resistance. Eritoran works by competing with LPS for the hydrophobic pocket in MD-2, preventing dimerization of TLR4-MD2 complexes (43). TAK-242 (Takeda Pharmaceuticals) is a small-molecule TLR4 inhibitor that binds selectively to Cys747 in the intracellular TIR domain of TLR4, preventing the association of TLR4 with its adapter molecules (44). We reported that TAK-242 protects muscle cells in vitro and rats in vivo from lipid- and LPS-induced insulin resistance (14, 45). Like eritoran, the effects of TAK-242 have been studied primarily with i.v. administration; whether this or other TLR4 antagonists could be administered orally for chronic treatment of insulin resistance remains undetermined.

This study has some limitations. First, the sample size is relatively small. Future studies with a larger sample would be helpful to further delineate the role of TLR4 in human insulin resistance. Second, the lipid infusion led to a modest (albeit significant) decrease in insulin sensitivity, which may have masked the effect of eritoran. Last, we did not test the effect of eritoran on individuals with obesity receiving lipid infusion to evaluate whether eritoran provides some protection when lipotoxicity is exacerbated.

In summary, TLR4 inhibition with eritoran did not improve glucose metabolism parameters in humans, which does not support a role for TLR4 in insulin resistance. Future studies with TLR4 inhibitors with different pharmacodynamic and pharmacokinetic properties may help to further clarify the role of TLR4 in insulin resistance in humans.

## Methods

### Study participants.

Through local advertisements, we recruited lean individuals (BMI <25) and individuals with obesity (BMI 30–37) for protocols I and II, respectively. All participants were sedentary and had stable body weight (±1.5%) for over 3 months before entering the study. All participants underwent a medical history assessment, physical examination, screening laboratory tests, and a 75 g oral glucose tolerance test to document normal glucose tolerance. Individuals were excluded if they had a history of inflammatory, hematologic, pulmonary, cardiovascular, hepatic, kidney, gastrointestinal, or neurologic disease or were taking any drug known to alter glucose metabolism, including antidiabetic agents.

### Study intervention.

Within 30 days of the screening tests, participants were admitted to the Bartter Research Unit of the Audie L. Murphy VA Medical Center. Following a double-blinded, randomized crossover design for protocol I, participants were studied on 3 occasions separated by a washout period of 4–8 weeks to receive: (a) saline+vehicle; (b) Intralipid+vehicle; or (c) Intralipid+eritoran. Saline and 20% Intralipid were obtained from Baxter and infused continuously via an antecubital vein at 30 mL/h for 72 hours. Eritoran (gift from Eisai) was administered at 12 mg every 12 hours for 72 hours. The drug was diluted in a vehicle solution (100 mL dextrose 5% in water [D5W]) and infused over 4 hours via the contralateral antecubital vein. For protocol II, which was also a double-blinded, randomized crossover study, the participants received eritoran (12 mg) or vehicle every 12 hours for 72 hours. During hospitalization, the participants remained ambulatory and consumed a weight-maintaining diet with 3 meals daily (accounting for the lipid administration) based on their habitual energy intake and food preferences. Eritoran was well tolerated and did not cause toxicity or any serious adverse event. Transient, mild-to-moderate phlebitis was observed at the eritoran infusion site in 4 individuals from protocol I and in 3 individuals from protocol II.

### Insulin clamping.

At the end of the interventions in both protocol I and protocol II, insulin sensitivity with a 80 mU/m^2^/min hyperinsulinemic euglycemic clamp was measured (46). Clamps started between approximately 7 am and 8 am, with participants in the fasting state. Peripheral insulin sensitivity was reported as the M value during the last 30 minutes of the clamp. Plasma glucose, insulin, and FFA concentrations before and during the last 30 minutes of the clamp are shown in Supplemental Tables 1 and 2.

### Laboratory analyses.

Plasma glucose was measured using an Analox analyzer and hemoglobin A1c (HbA1c) using a DCA2000 analyzer (Bayer). Plasma insulin was measured by radioimmunoassay (Diagnostic Products). The HOMA-IR index was calculated as (FPI × FPI)/22.5. Plasma FFA levels were measured using a kit from FUJIFILM Wako Chemicals. Plasma LPS levels were determined using a limulus amoebocyte lysate (LAL) assay (Lonza). Plasma TNF-α levels were measured with an ELISA kit from R&D Systems. A baseline sample for TNF-α measurement was missing for 1 person in protocol II.

### Ex vivo LPS stimulation test.

Peripheral blood was treated with 10 ng/mL LPS (MilliporeSigma) for 3 hours, and plasma was assayed for the TNF-α concentration. One individual from protocol I and 2 individuals from protocol II had insufficient amounts of blood collected for ex vivo stimulation testing with LPS.

### Statistics.

All data are represented as the mean value of each group ± SEM. Statistical tests are described in the figure legends. Differences between groups were determined using a paired Student’s *t* test, a Wilcoxon signed-rank test, and a 1-way or 2-way ANOVA, where appropriate. Statistical significance testing was 2-sided and assumed at a *P* value of less than 0.05.

### Study approval.

This study was approved by the IRB of the University of Texas Health Science Center at San Antonio and was registered at ClinicalTrials.gov (NCT02321111 and NCT02267317). All study participants provided written informed consent. The protocol was carried out under investigational new drug (IND) application number 123955 (sponsor: NM).

## Author contributions

NM designed the study. NM, NS, CC, VG, JG, SE and HL contributed to data acquisition, data analysis, and data interpretation. NM and HL wrote the first draft of the manuscript. All authors contributed to the revision of the manuscript.

## Supplementary Material

Supplemental data

Trial reporting checklists

ICMJE disclosure forms

## Figures and Tables

**Table 1:**
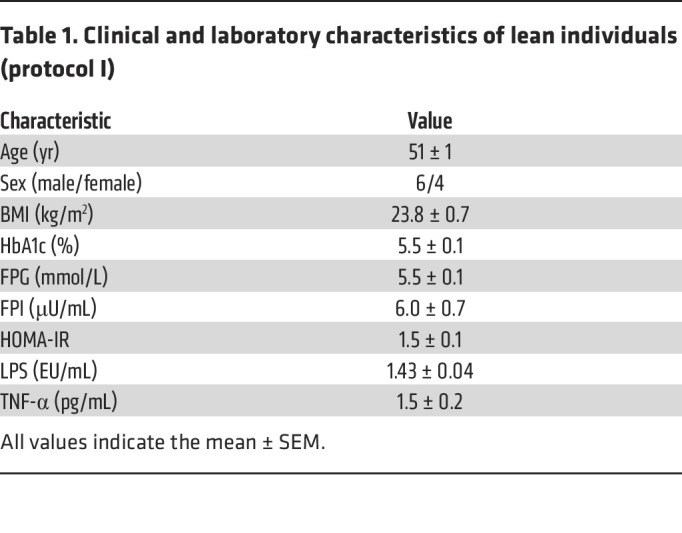
Clinical and laboratory characteristics of lean individuals (protocol I)

**Table 2:**
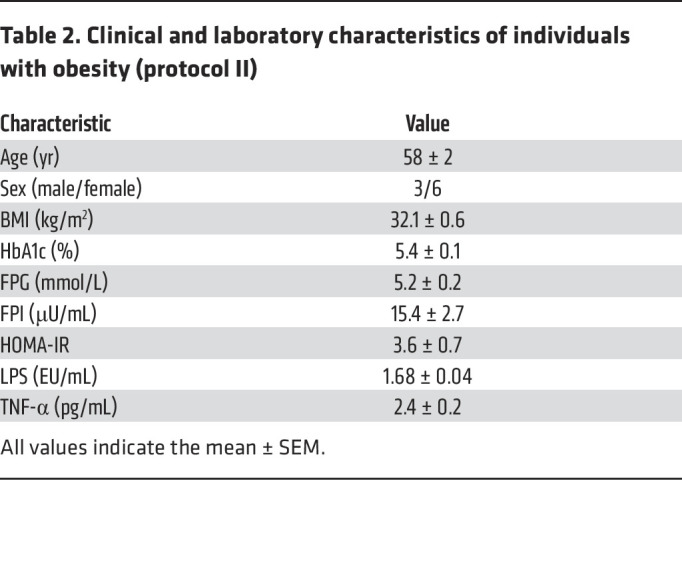
Clinical and laboratory characteristics of individuals with obesity (protocol II)

## Version 1 09/06/2022

In-Press Preview

## Version 2 11/01/2022

Electronic publication
