# Supplementary material for: Effect of acute TLR4 inhibition on insulin resistance in humans
Source: J Clin Invest. 2022 Nov 1;132(21):e162291. doi: 10.1172/JCI162291 (PMC9621129; doi:10.1172/JCI162291)
Supplement: Supplemental data [file jci-132-162291-s135.pdf]

Effect of acute TLR4 inhibition on insulin resistance in human subjects

Hanyu Liang, Nattapol Sathavarodom, Claudia Colmenares,

Jonathan Gelfond, Sara Espinoza, Vinutha Ganapathy, Nicolas Musi

SUPPLEMENTARY MATERIALS

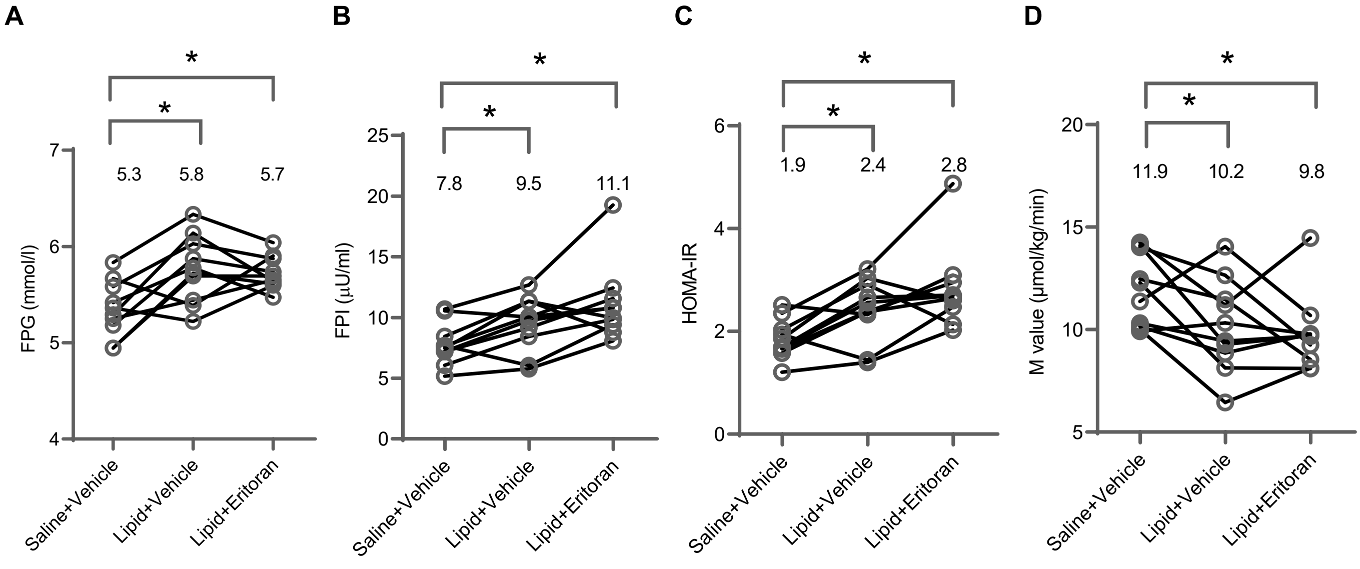

Figure S1. Effect of lipid and eritoran on metabolic parameters in lean subjects. Fasting blood glucose (FPG) (A), fasting plasma insulin (FPI) (B), HOMA insulin resistance index (HOMA-IR) (C), and insulin-stimulated glucose metabolism (M values) (D) were determined following saline+vehicle, lipid+vehicle, and lipid+eritoran infusions. n=10. Mean values of each group are shown above individual data points. Data were compared using one-way ANOVA followed by Holm-Šídák's multiple comparisons test. * $P < 0.05$.

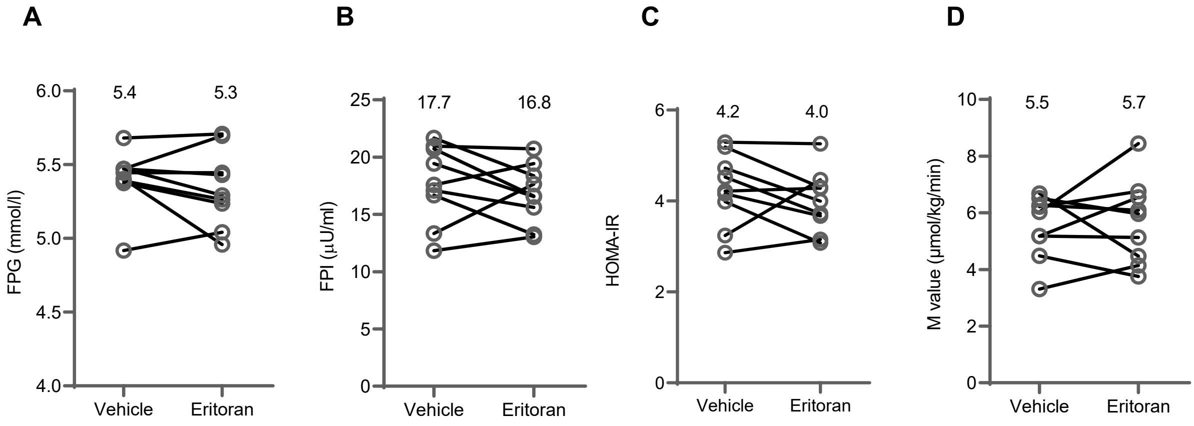

Figure S2. Effect of eritoran on metabolic parameters in obese subjects. Fasting blood glucose (FPG) (A), fasting plasma insulin (FPI) (B), HOMA insulin resistance index (HOMA-IR) (C), and insulin-stimulated glucose metabolism (M values) (D) were determined following vehicle or eritoran administration. n=9. Mean values of each group are shown above individual data points. Comparisons were made using paired t-tests or Wilcoxon signed-ranked test.

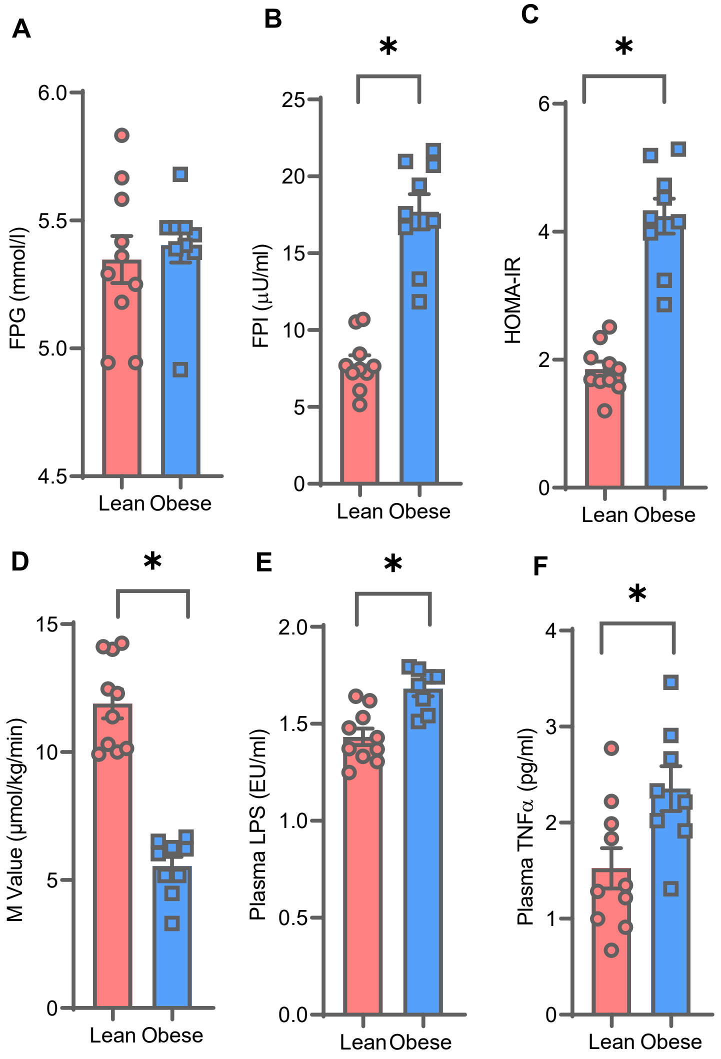

Figure S3. Comparison of metabolic parameters in lean and obese subjects. FPG (A), FPI (B), HOMA-IR (C), M value (D), plasma LPS (E) and TNFα levels (F) were determined in lean and obese subjects, following saline+vehicle (Protocol I) and vehicle administration (Protocol II), respectively. All data are the mean ± SEM of data obtained from 10 lean and 9 obese subjects. FPG: Fasting plasma glucose; FPI: fasting plasma insulin; M value: insulin-stimulated glucose metabolism; Data were compared using an unpaired t-test. * $P < 0.05$.

Table S1. Plasma glucose, insulin and FFA concentrations before and during the insulin clamp – Protocol I

Parameters		Saline+Vehicle	Lipid+Vehicle	Lipid+Eritoran
**FFA (µmol/l)**	**Basal**	513 ± 65	481 ± 47	537 ± 26
	**Clamp**	52 ± 11	157 ± 36*	138 ± 8*
**Insulin (µU/ml)**	**Basal**	7.8 ± 0.5	9.5 ± 0.7*	11.1 ± 1.0*
	**Clamp**	183.1 ± 10.5	178 ± 9.3	180.4 ± 9.1
**Glucose (mmol/l)**	**Basal**	5.3 ± 0.1	5.8 ± 0.1*	5.7 ± 0.1*
	**Clamp**	5.5 ± 0.1	5.5 ± 0.1	5.4 ± 0.1

All values are mean ± SEM. n=10. * P <0.05 compared to saline+vehicle. Comparisons made with repeated measures ANOVA followed by Holm-Šídák's multiple comparisons test.

Table S2. Plasma glucose, insulin and FFA concentrations before and during the insulin clamp – Protocol II

Parameters		Vehicle	Eritoran
FFA (µmol/l)	Basal	536 ± 35	605 ± 78
	Clamp	48 ± 7	52 ± 7
Insulin (µU/ml)	Basal	17.7 ± 1.1	16.8 ± 0.9
	Clamp	239.2 ± 16.8	252.8 ± 14.2
Glucose (mmol/l)	Basal	5.4 ± 0.1	5.3 ± 0.1
	Clamp	5.4 ± 0.0	5.5 ± 0.1

All values are mean ± SEM. n=9. Treatments compared with a paired t-test.
